# Supplementary material for: LncRNA WDR11-AS1 Promotes Extracellular Matrix Synthesis in Osteoarthritis by Directly Interacting with RNA-Binding Protein PABPC1 to Stabilize SOX9 Expression
Source: Int J Mol Sci. 2023 Jan 3;24(1):817. doi: 10.3390/ijms24010817 (PMC9820994; doi:10.3390/ijms24010817)
Supplement: Supplementary file 1 [file ijms-24-00817-s001.zip › supplementary Table S1.pdf]

Supplementary Table S1. Sequence of siRNAs

siRNA	sense (5′-3′)	antisense (5′-3′)
WDR11-AS1 si-1	CCAGACUGAAUGAAGUGAATT	UUCACUUCAUUCAGUCUGGTT
WDR11-AS1 si-2	UGAAUGAAAUUGCCAGACUTT	AGUCUGGCAAUUUCAUUCATT
WDR11-AS1 si-3	GAAAUUGCCAGACUGAAUGTT	CAUUCAGUCUGGCAAUUUCTT
PABPC1 si-1	GCCUGCCUUAAGUGUGAAATT	UUUCACACUUAAGGCAGGCTT
PABPC1 si-2	GUAGGCAACAUAUUCAUUATT	UAAUGAAUAUGUUGCCUACTT
PABPC1 si-3	CACCUCACUAACCAGUAUATT	UAUACUGGUUAGUGAGGUGTT
si-NC	UUCUCCGAACGUGUCACGUTT	ACGUGACACGUUCGGAGAATT

Notes: WDR11-AS1 WDR11 divergent transcript, PABPC1 polyadenylate-binding protein cytoplasmic 1, si-NC negative control siRNA
