# Supplementary material for: LncRNA WDR11-AS1 Promotes Extracellular Matrix Synthesis in Osteoarthritis by Directly Interacting with RNA-Binding Protein PABPC1 to Stabilize SOX9 Expression
Source: Int J Mol Sci. 2023 Jan 3;24(1):817. doi: 10.3390/ijms24010817 (PMC9820994; doi:10.3390/ijms24010817)
Supplement: Supplementary file 1 [file ijms-24-00817-s001.zip › supplementary Table S2.pdf]

Supplementary Table S2. Primers used for RT-qPCR

Gene	Forward primer (5′-3′)	Reverse primer (5′-3′)
COL Ⅱ	TGGACGATCAGGCGAAACC	GCTGCGGATGCTCTCAATCT
ACAN	GGCATTTCAGCGGTTCCTTCTC	AGCAGTTGTCTCCTCTTCTACGG
MMP3	AGTCTTCCAATCCTACTGTTGCT	TCCCCGTCACCTCCAATCC
MMP13	AATATCTGAACTGGGTCTTCCAAAA	CAGACCTGGTTTCCTGAGAACAG
ADAMTS4	CTACCTGACTGGCACCATCAAT	CCGATATTGTAACACGCCTAAC
ADAMTS5	GACCGATGGCACTGAATGTAGGC	TCTCCTCCACATACTCCGCACTTG
WDR11-AS1	ACCATGCAAACCACGTTCAC	TGCCCTGGATACACTTGCTG
PABPC1	CACCAGATACCAGGGTGTTAAT	GCCACAATTCTACCGTTCATTT
SOX9	GACAGCCCCCTATCGACTTC	CAAACTCGTTGACATCGAAGG
SOX6	GACCAGTGTTACTTTTGGAACC	TTTCTCCAAGAAGTTCACTGGT
β-ACTIN	AGTTGCGTTACACCCTTTCTTG	TCACCTTCACCGTTCCAGTTT
GAPDH	CACCCACTCCTCCACCTTTG	CCACCACCCTGTTGCTGTAG
U6	CTCGCTTCGGCAGCACA	AACGCTTCACGAATTTGCGT

Notes: COL Ⅱ type II collagen, ACAN aggrecan, MMP matrix metalloproteinase, ADAMTS a disintegrin and metalloproteinase with thrombospondin motifs, WDR11-AS1 WDR11 divergent transcript, PABPC1 polyadenylate-binding protein cytoplasmic 1, SOX SRY-related high-mobility group box, β-ACTIN actin beta, GAPDH glyceraldehyde phosphate dehydrogenase, U6 small nuclear RNA U6
